# The Health of the Dentists

**Published:** 1891-03

**Authors:** Howard Van Antwerp

**Affiliations:** Mt. Sterling, Ky.


### The Health of the Dentist.

BY HOWARD VAN ANTWERP, D.D.S., MT. STERLING, KY.

The popular idea seems to be that the practice of dentistry is very much harder on the constitution of those engaged in it than other professions.

A casual glance at the situation will almost always result in the above conclusion. Why is it?

The observer notes on entering the office, first of all, the close, musty, small, peculiar to all ill-ventillated dental and medical offices. Things look dingy and dust lies, a week old perhaps, on all unusued articles. He is seated in the chair, which by the way, has much to do with the health of the man working at it. Here he soon finds the source of some of the disagreeable odor, so marked on first entry, viz.: the cuspidor. The operator, wholly regardless of the patient's comfort or his own physique, sprawls himself out and goes to "goughing." The patient, already scared half to death and nearly sick, winches. Result—a more savage attack on the carious center and a more pronounced winch. This goes on from bad to worse until the dentist gets "rattled" and can hardly restrain his excited feelings until the fillings are in, and the muchly used-up patient dismissed. He then retires to "cuss" his fate or the labratory "cub" or worse still, seeks to soothe his anger with a good (?) cigar.

Is it any wonder that that patient should think it would be trying to be a dentist and undergo similar experiences day in and day out for years. It is not the physical exertion, but it is this mental excitement oft repeated, that wears dentists out. Habit is strong, and before one is aware of it he is called "crabbed and cross" by his patients. A cheerful bearing and a pleasant way of dealing with "bad" patients, go far toward the preservation of the operator's health.

Too great a love for the almighty dollar has much to do with the physical standing of the profession. It leads its victim to working early and late, to the taking of his meals at irregular hours and in a hasty manner, and prevents him from indulging in any diversion that *might* cause him to loose a dollar or two, but which would, if taken, prove of incalculable benefit to him in the end. A minute's sober reflection will show any of us wherein we are going wrong. Why will we continue doing that which we know to be injurious in its effects.

Dietitically, each must be his own judge. Generally speaking, we all eat too much and take too little time doing it. We do not adapt our diet, either to our individual needs or to reason, common sense should decide the former and nature's plan is a very good one in the latter. Many continue their winter diet of meats, fats, rich milk and butter through the summer, and then wonder why they are so uncomfortable in the hot season. Stimulents and narcotics, mild or strong, are to be unreservedly condemned.

A cheerful office, well ventillated and clean can be secured by all, and will go far toward securing that " tone " that a few can only obtain by expensive office appointments.

Personal cleanliness is a very important factor, not only in the health of the dentist, but also in his material welfare. This costs nothing and the delightful self-satisfaction one feels more than pays for the time expended.

A comparatively easy, upright position at the chair is easily maintained with most modern chairs. There is no sensible reason for stoop-shoulders or lateral spinal curvatures, especially should no position be taken which cramps the abdominal viscera and so interferes with their normal action. Young operators may correct the decided elevation of left shoulder by sleeping with right shoulder the highest. Don't get " down in front " of the patient's nose when working. It is a robust operator, with a phenomenal pair of lungs, that can persist in some of the positions taken in the operating room and come out unscathed in ten years.

The art of leaving your professional self behind when you leave your office is of no mean importance. This can only be acquired by long practice and thorough occupation in some pleasant recreation, bodily or mental, nine months in the year, in almost any State, are suitable for out-door exercises. Out-door exercise is what the dentist needs, combining as it does, pure air, sunlight for the body and exhileration for the mind. The need of the first two is plainly seen at a glance. The third, exhileration, is no less valuable. Many diseases, often called incurable, of the digestive tract depend on some functional derangement. Exercise, to correct these affections must work on the cause. A keen sense of enjoyment in out-door recreation is the most potent tonic at our command. On the other hand, "fatigue" must be carefully avoided. To make "work" of recreation and to carry it to the point of exhaustion, only serves to debilitate and break down. Nothing but harm can come from this over-exertion. What then is the exercise suited to the dentist? Horseback riding is too expensive for ordinary mortals. A good saddle horse is very hard to get and when secured costs the interest on a small fortune to keep. A timid rider rarely becomes a confident one, and without confidence and ease there can be no enjoyment.

Walking is very good but soon grows monotonous. One gets the air but rarely the bodily or mental excitement.

Lawn tennis, in its season, is nearly the thing. Here we have a form of exercise that is not liable to merge into work and combines fresh air and sunlight with social pleasure. Skill in the game can be cultivated indefinately and the interest in it rarely lessens, especially if the interested party be "single" and has two or three young lady friends who play. Truly it is called a "love" game.

These and many other games and sports will easily find able champions in the ranks of the profession, but above them all we desire to place bicycling, or "wheeling" as it is called by the initiated. This, it seems to us, is the *ne plus ultra* of out-door recreations, combining as it does, in a most perfect degree, all the desirable features of such, and yet almost wholly without any of the drawbacks attendant on other forms.

Generally speaking, there are three classes of wheels in vogue; the high crank machine or "ordinary," the little-wheel-in-front high machine and the safeties. The first class are the swiftest and most graceful appearing, and enable their riders to approach nearest to that "bird flight" so often spoken of in cycling literature. They are too, the most dangerous, only the most confident riders using them. The second class obviates the greatest danger of the ordinaries, viz.: "header." The Star is the great exponent of this class. The writer rides one, and for all-'round use would exchange it for no other. The machine, practicable for the use of the majority of dentists, is the modern "safety," a creation of the last decade. Originally designed for professional and elderly men, its merits have placed it on a par with other wheels, if, indeed, not above them. This wheel combines nearness to ground with the speed of a high wheel. Positively no danger, unless ridden recklessly. Takes up little room and adds to, rather than detracts from, the appearance of an office. The old and young, ladies as well as gentlemen can and do ride them. "Pap" Ruff, of Richmond, Ky., is 65 years old, rides a safety and is one of the most enthusiastic wheelmen in that State, while Chicago has nearly 1000 lady riders of safeties.

The primary cost is comparatively small, care and repair cost practically nothing; is easily mastered by the timid as is demonstrated by its popularity with the ladies. A tandem—two seated—introduces a social feature not to be lost sight of, lady usually riding the front seat.

Always ready for a five minute run or an all day's tour. With a fifteen minute spin you are carried into the country in almost any direction. There you bowl along at your pleasure, following the windings of some creek or river road. Rounding the basis of hills and projecting points, each succeeding curve furnishes you with new and attractive views from nature's scenery folio. The air is deliciously cool and moist, and possibly fragant with the perfume of the wild crab-apple or cherry. The man is indeed a poor creature who cannot forget all earthly care at such a time.

Or, perhaps, you prefer to take a high ridge road and fill the remotest air cell in your lungs with that high, dry air, so healthful that one feels almost as though he had taken some wonderful elixir. In his exultation, he wonders how a body, feeling so bouyant and strong, can ever become the prey of disease.

On the return trip you seek out, if you be not too prosey, some shady, flower-scented lone, and chase the moonbeams as they play through the branches of the arching trees above. Truly this *is genuine* pleasure and only such as can be experienced by the cyclist.

"Through every fiber of my brain,
Through every nerve, through every vein,
I feel the electric thrill, the touch
Of life, that seems almost too much."

With joy I mount my silent wheel,
And over hill and valley steal,
Thrilled with the thought that unto me
Is given boundless liberty.

"I hear the wind among the trees
Playing celestial symphonies;
I see the branches downward bent
Like keys of some great instrument."

And over me unrolls on high
The splendid scenery of the sky,
Where through a sapphire sea the sun
Sails like a golden galleon.

"Filled with a sense of keen delight
I ride and ride, until the night
Darkens about me. Home I fly,
Glad I can thus my cares defy."

There is something in cycling that is satisfying to the soul. It affords free communion with nature and inspires us with a sense of gratitude for Divine goodness, gives healthful exercise to the body, and restful exhileration to the brain. Does it not meet all the requirements of the case? Is it not the exercise for the conservation of the health of the dentist?

### A Temperance Proverb.

A German proverb says: "Intemperance drives reason out of the head, money from the pocket, the elbows through the sleeves and health from the body.

